# Assessment of Nutritional Status and Energy Availability of Indian Boxers

**DOI:** 10.7759/cureus.63730

**Published:** 2024-07-03

**Authors:** Neelam Rathod, Suneeta Chandorkar

**Affiliations:** 1 Foods and Nutrition, The Maharaja Sayajirao University of Baroda, Vadodara, IND

**Keywords:** low energy availability, energy availability, nutritional status, indian boxers, indian athletes

## Abstract

Background: Inadequate dietary intake and low energy availability (LEA) negatively impact athlete's health, recovery, and performance outcomes. Currently, there is no Indian study that assessed the energy availability (EA) of Indian boxers and studied the contributing factors to LEA. This study was undertaken with the objectives of assessing the EA of Indian boxers (lightweight category) and correlating it with contributing factors such as anthropometric measurements, body composition, dietary intake, nutrient intake, and exercise energy expenditure.

Methods: This descriptive cross-sectional study recruited 30 state-level boxers of the lightweight category (females: nine) residing in Mumbai, Maharashtra, India, using purposive sampling. Body composition was assessed using a body composition monitor. Anthropometric measurements were assessed using standard methods. Three-day exercise energy expenditure was calculated. Three-day dietary intake was assessed, which included two training days and one rest day using the three-day multiple-pass method. Dietary intake assessment included intake of all the meals, snacks, sports foods, sports, and dietary supplements. EA was calculated and participants were classified as moderate EA (30-45 kcal/kg FFM/day) and low EA (< 30 kcal/kg FFM/day). Descriptive and inferential statistical tests were conducted using Statistical Product and Service Solutions (SPSS, version 23; IBM SPSS Statistics for Windows, Armonk, NY).

Results: Male boxers had a mean age of 18.43±0.93 years, and females had a mean age of 18.44±0.88 years. Among boxers, 36.67% (n=11) of participants were underweight, 46.67% (n=14) had a normal BMI, and 16.67% (n=5) were overweight. The mean body fat (%) for boxers was 13.11±1.8. Participants had insufficient consumption of different food groups before and after the energy intake adjustment. Processed packaged food consumption was popular among the participants. Various kinds of sports foods and supplements were consumed by a few athletes. Dietary supplements were not common among participants. The nutrient intake of the participants was inadequate. Participants could not fulfill energy/kg/day, carbohydrate/kg/day, protein/kg/day, and fat/kg/day requirements by 29%, 36.2%, 34.8%, and 23.9%, respectively. Iron and calcium intake was inadequate among female participants by 1.4% and 12.8%, respectively. Among male boxers, the mean EA was 29.71±3.74 kcal/kg FFM/day, and the mean EA of female boxers was 28.3±3.95 kcal/kg FFM/day. Around 66.67% of boxers in the present study had LEA, while the rest had reduced EA. EA had a moderately significant inverse relationship with fat-free mass (%). Energy intake was significantly related to EA and total body muscle % significantly moderated that relationship. The influence of fat-free mass (%), EEE (kcal), and energy intake (kcal) on the EA (kcal/kg FFM/day) was significant using multiple linear regression analysis.

Conclusions: The majority of the participants could not meet the food intake and nutrient recommendations. The majority of the participants had LEA. The higher the energy and nutrient intake, the higher the EA in the present study. EA had a moderately significant inverse relationship with fat-free mass (%) and exercise energy expenditure.

## Introduction

Nutrition is the study of foods and nutrients and their effects on human health, growth, and development. Sports nutrition applies nutrition principles to sports to optimize performance. Optimal diet strategies can enhance an athlete’s sports performance, training adaptations, recovery, and general health. An optimal diet may be defined as one in which the supply of required nutrients is adequate to cover energy expenditure, tissue maintenance, repair, and growth [1].

To fulfill the nutrient requirements of athletes as compared to sedentary individuals, it is important to increase the quantity of nutrient-dense food groups such as carbohydrate-rich foods like cereals and millets, roots and tubers, protein-rich foods like pulses and legumes, milk and milk products, animal-based foods, fat-rich foods like nuts and seeds, antioxidants rich foods such as whole grains, and variety of fruits and vegetables and reduce the consumption of processed snacks (both vegetarian and non-vegetarian), midnight snacking, frequent dining, out and take away foods. This will also aid in meeting the recommendation for micronutrients. Inadequate dietary intake can lead to low energy availability (EA), which affects both male and female athletes. Low EA (LEA) occurs when an athlete's energy intake is insufficient to support the body's necessary functions for optimal health and performance due to exercise energy expenditure [2]. EA is defined as the amount of dietary energy available to sustain physiological function after subtracting the exercise energy expenditure [3]. Both male and female elite athletes are at risk of LEA [4]. The female and male athlete triad and relative energy deficiency in sport (RED-S) are based on LEA. The disorder develops when an athlete consumes insufficient calories to fuel their physical activity, which compromises physiological functions and causes various endocrine issues [5]. Low EA risk is greater in athletes of aesthetic, weight-sensitive, and endurance-based sports than in team sports [6]. The conventional EA equation is as follows: EA (kcal/kg FFM/day) = (EI(kcal/day) − EEE(kcal/day))/FFM (kg), where EI = energy intake and EEE = exercise energy expenditure. An EA of < 30 kcal/kg FFM/day is typically defined as clinically low EA [7].

Boxing is a combat sport with high pace and intensity. It requires both aerobic and anaerobic fitness, as well as considerable skill [8]. There are both amateur and professional boxing competitions. Boxers are grouped into weight divisions for the competitions and weigh-ins are held before the competition. Boxers do engage in rapid weight loss practices in the hours and days before the weigh-in. Extreme dehydration by vomiting, starvation, laxatives, and the use of diuretics significantly decrease the body weight before weigh-in for competition. These practices can be dangerous to health and are strongly discouraged [8]. Both chronic and acute weight loss practices are seen in the training phase of boxers and were in a state of LEA during the entire training period in the previous study [9]. There are no studies reported with respect to the ES of Indian boxers. Hence, it is critical to assess the boxer’s food intake along with the intake of sports foods, sports supplements, and dietary supplements at regular intervals.

Sports nutrition assumes critical importance because, long before deficiency symptoms start appearing, physical performance declines [1]. Since well-chosen nutrition strategies are important for success in sports and can affect performance, it is important to assess a complete picture of the nutrition strategy followed by athletes. Currently, there is no Indian study that assessed the EA of Indian boxers and studied the contributing factors to LEA. This study was undertaken with the objectives of assessing the EA of Indian boxers (lightweight category) and correlating it with contributing factors such as anthropometric measurements, body composition, dietary intake, nutrient intake, and exercise energy expenditure.

## Materials and methods

Study population

A total of 30 boxers (lightweight) residing in Mumbai, Maharashtra, India, gave written consent to participate in this cross-sectional study. The sample was purposively selected after fulfilling the inclusion criteria. The athletes who fulfilled the following criteria were included in the study: (1) men and women athletes, (2) 18 years and above, (3) competed at the state level or higher in their chosen sport, (4) training continuously for at least 24 months, (5) should be training for at least four hours a day, and (6) training six days a week. The participants of the following criteria were excluded from the study: (1) exercisers, (2) paralympics, (3) athletes in the competitive and off-season, (4) currently having some medical conditions - pathological or physiological, and (5) not willing to participate. The present study was approved by the Institutional Ethics Committee for Human Research (IECHR), Faculty of Family and Community Sciences, The Maharaja Sayajirao University of Baroda. The study has been allotted the ethical approval number IECHR/FCSc/PhD/2021/126.

Study design

Background information was collected using a semi-structured questionnaire, followed by anthropometric measurements of the participants and body composition assessments. Three-day exercise energy expenditure was calculated. Three-day dietary intake was assessed, which included two training days and one rest day. Dietary intake assessment included intake of all the meals, snacks, sports foods, sports supplements, dietary supplements, and water intake. EA was calculated. Tools and techniques used in the present study have been described in detail in Table 1.

**Table 1:** Tools and techniques

Sr. No.	Parameter	Method	Tools and Techniques/Formulas
1	Body Height (cm)	Assessment	Non-stretchable Fiberglass tape
2	Body Weight (kg)	Assessment	Body Composition Monitor- Karada Scan, model HBF-362, Omron, Osaka, Japan
3	BMI (kg/m^2^)	Calculation	Quetelet index: BMI = Weight(kg)/Height(m^2^),WHO Asian cutoff points
4	Body Composition (Body Fat %,Body Muscle %, Visceral Fat %)	Assessment	Body Composition Monitor- Karada Scan, model HBF-362, Omron, Osaka, Japan
5	Dietary Intake	Interview- Face to Face + Calculation	Standardized household measures- Measuring Spoon and Cup Set , 3 days 24-hour dietary recall-3 days Multiple-Pass Method (2 training days+1 rest day)[10] , Food frequency questionnaire(semi-structured) , Ntutive application (Fitterfly Healthtech Pvt. Ltd., Navi Mumbai, India) for nutrient calculation (macronutrients, micronutrients and others) which uses the Indian Food Composition Table, ICMR-NIN,2017
6	Exercise Energy Expenditure (Kcal/min)	Interview- Face to Face + Calculation	Mean of 3-day exercise energy expenditure, MET value was used from the Compendium of Physical Activities,2011 [11]
7	Energy Availability (Kcal⋅kg FFM⋅day^−1^)	Interview- Face to Face + Calculation	EA(kcal⋅kg FFM⋅day^−1^)=EI(kcal) -EEE(kcal) /FFM(kg)[7]

Statistical analysis

The data were analyzed using Statistical Product and Service Solutions (SPSS, version 23; IBM SPSS Statistics for Windows, Armonk, NY). Descriptive statistics such as the mean, median, standard deviation, and range of food intake, gender-wise, before and after energy adjustment were assessed. Nutrient intake and the percentage of nutrient recommendations and micronutrient recommendations (EAR) fulfilled by gender were also assessed. The frequency of consumption of packaged processed foods, sports foods, sports supplements, and dietary supplements was assessed using percentages. Normal distribution was tested using Shapiro-Wilk. If the normal distribution of data was significant, a t-test was used. If the normal distribution of data was not significant, the Mann-Whitney U test was used instead of the t-test. The difference was significant at ≤0.05 and highly significant at ≤0.01 using the t-test/Mann-Whitney U test. Pearson’s correlation test was carried out between EA and various contributing factors such as nutrient intake, food intake, body composition, and exercise energy expenditure. A multiple linear regression analysis was carried out to assess the influence of various parameters on EA. Moderation analysis was used to assess the relationship between energy intake and EA moderated by body composition.

## Results

Descriptive characteristics

A total of 30 Indian boxers of the lightweight category residing in Mumbai city, Maharashtra state, India, participated in this study. Around 70% of boxers were male (n=21). Male boxers had a mean age of 18.43±0.93 years, and females had a mean age of 18.44±0.88 years. Around 96.67% (n=29) of boxers were undergraduates, and 3.33% (n=1) graduated. The male boxers trained for 5.9±0.83 years, and the females trained for 5.67±0.5 years.

Dietary intake without energy adjustment

Descriptive statistics such as mean, median, standard deviation, and range of intake of various food items by boxers and inferential statistics such as t-test/Mann-Whitney U test and effect size for gender-wise differences in intake of food items are presented in Table 2. The mean intake of carbohydrate-rich food items per day without energy adjustments such as cereals and grains, roots and tubers, fruits, processed fruits, and milk and milk products were 351.0±24.1 g, 96.3±50.1 g, 156.6±76.2 g, 14.5±9.1 g, and 333.3±189.5 mL, respectively. The mean intake of protein-rich food items daily without energy adjustments such as pulses and legumes, eggs, and poultry was 38.0±20.7 g, 115.0±51.1 g, and 130±61.0 g. The mean intake of green leafy vegetables and other vegetables per day was 35.6±30.3 g and 183.3±29.6 g, respectively. The mean intake of added sugar per day was 40.7±3.7 g. The mean intake of solid fats and cooking oil per day was 2.4±5.3 g and 39.5±4.1 mL, respectively. There were significant differences in the intake of cereals and grains, other vegetables, and added sugar based on gender. The effect size (r=0.85) of the gender-wise differences in the intake of cereals, grains, and other vegetables was strong in the present study.

**Table 2:** Intake of various food items according to gender G: Gender, n: Frequency, %: Percentage, Mdn.: Median, Min.: Minimum, Max.: Maximum, S.D.: Standard Deviation, p≤0.05: Significant, p≤0.01: Highly Significant

Food items	Descriptive Statistics						Inferential Statistics	
	G	n (%)	Mdn.	Min.	Max.	Mean ± S.D.	t-test/Mann-Whitney U test	Effect Size
Cereals and grains (g/day)	M	21 (70)	360	330	375	358.5 ± 13.3	t(28) = 2.15, p = 0.06	d=0.85
	F	9 (30)	330	300	375	333.3 ± 34.1		
	T	30 (100)	360	300	375	351.0 ± 24.1		
Pulses and legumes (g/day)	M	21 (70)	30	30	90	38.5 ± 23.5	U = 89, p = 0.82	r=0.07
	F	9 (30)	30	30	60	36.6 ± 13.2		
	T	30 (100)	30	30	90	38.0 ± 20.7		
Green leafy vegetables (g/day)	M	21 (70)	50	0	100	39.0 ± 31.9	t(28) = 0.93, p = 0.36	d=0.37
	F	9 (30)	50	0	50	27.7 ± 26.3		
	T	30 (100)	50	0	100	35.6 ± 30.3		
Other vegetables (g/day)	M	21 (70)	200	100	200	190.4 ± 25.5	t(28) = 2.14, p = 0.04	d=0.85
	F	9 (30)	175	100	200	166.6 ± 33.0		
	T	30 (100)	200	100	200	183.3 ± 29.6		
Roots and tubers (g/day)	M	21 (70)	130	0	130	102.3 ± 49.1	t(28) = 1.01, p = 0.32	d=0.40
	F	9 (30)	100	0	130	82.2 ± 52.3		
	T	30 (100)	130	0	130	96.3 ± 50.1		
Fruits (g/day)	M	21 (70)	200	0	200	171.4 ± 62.3	U = 70.5, p = 0.29	r=0.25
	F	9 (30)	200	0	200	122.2 ± 97.1		
	T	30 (100)	200	0	200	156.6 ± 76.2		
Processed fruit products (g/day)	M	21 (70)	20	0	25	15.0 ± 8.9	t(28) = 0.45, p = 0.655	d=0.18
	F	9 (30)	20	0	20	13.3 ± 1.0		
	T	30 (100)	20	0	25	14.5 ± 9.1		
Eggs (g/day)	M	21 (70)	150	0	150	126.1 ± 46.4	t(28) = 1.91, p = 0.06	d=0.45
	F	9 (30)	100	0	150	88.8 ± 54.6		
	T	30 (100)	150	0	150	115.0 ± 51.1		
Poultry (g/day)	M	21 (70)	150	0	200	142.8 ± 53.1	t(28) = 1.83, p = 0.07	d=0.73
	F	9 (30)	100	0	200	100 ± 70.7		
	T	30 (100)	150	0	200	130 ± 61.0		
Nuts (g/day)	M	21 (70)	15	0	20	11.9 ± 8.4	t(28) = -0.89, p = 0.38	d=0.36
	F	9 (30)	20	0	25	15.0 ± 9.3		
	T	30 (100)	15	0	25	12.8 ± 8.6		
Milk and milk products (mL/day)	M	21 (70)	250	0	750	357.1 ± 186.6	t(28) = 1.05, p = 0.30	d=0.42
	F	9 (30)	250	0	500	277.7 ± 195.4		
	T	30 (100)	250	0	750	333.3 ± 189.5		
Added sugar (g/day)	M	21 (70)	40	40	50	41.7 ± 3.4	t(28) = 2.49, p = 0.01	d=0.9
	F	9 (30)	40	30	40	38.3 ± 3.5		
	T	30 (100)	40	30	50	40.7 ± 3.7		
Cooking oil (mL/day)	M	21 (70)	40	30	50	39.5 ± 3.8	t(28) = -0.12, p = 0.90	d=0.05
	F	9 (30)	40	30	50	39.7 ± 5.0		
	T	30 (100)	40	30	50	39.5 ± 4.1		
Solid fats (g/day)	M	21 (70)	0	0	22.5	2.5 ± 5.8	t(28) = 0.13, p = 0.89	d=0.05
	F	9 (30)	0	0	10	2.2 ± 4.4		
	T	30 (100)	0	0	22.5	2.4 ± 5.3		

Dietary intake with energy adjustment

Dietary recommendations given in the Nutrition and Hydration Guidelines for Excellence in Sports Performance were followed to assess the adequacy of dietary data [1]. Descriptive statistics such as mean, median, standard deviation, and range of intake of various food items after energy adjustment by boxers and inferential statistics such as t-test/Mann-Whitney U test and effect size for gender-wise differences in intake of different food items are presented in Table 3. The mean intake of carbohydrate-rich food items with energy adjustments such as cereals and grains, roots and tubers, fruits, processed fruits, and milk and milk products were 142.2±9.7 g/1000 kcal, 38.8±20.5 g/1000 kcal, 62.3±30.7 g/1000 kcal, 5.9±3.7 g/1000 kcal, and 133.7±75.9 mL/1000 kcal, respectively. Participants exceeded the recommendation for cereals and grains and fruits, while intake of other carbohydrate-rich food items fell below the recommendations. The mean intake of protein-rich food items after energy adjustments such as pulses and legumes, eggs, and poultry were 15.7±9.60 g/1000 kcal, 45.9±20.5 g/1000 kcal, and 52.46±24.6 g/1000 kcal. Participants exceeded the recommendation for pulses and legumes and eggs, while intake of poultry fell below the recommendations. Only 35% of recommendations were fulfilled for green leafy vegetables, while the intake of other vegetables exceeded 38% of recommendations. The mean intake of added sugar was 16.6±2.5 g/1000 kcal. Participants exceeded the total intake of added sugar marginally. The mean intake of solid fat and cooking oil were 16.1±2.2 g/1000 kcal and 0.2 mL/1000 kcal, respectively. Participants exceeded the recommendation of cooking oil by 15%. Gender-wise differences in intake of cooking oil were significantly different.

**Table 3:** Intake of various food items after energy adjustment according to gender G: Gender, n: Frequency, %: Percentage, Mdn.: Median, Min.: Minimum, Max.: Maximum, S.D.: Standard Deviation, p≤0.05: Significant, p≤0.01: Highly Significant

Food Items	Descriptive Statistics								Inferential Statistics	
	G	n (%)	Mdn.	Min.	Max.	Mean ± S.D.	Recommendation (g)	% of recommendations fulfilled	t-test/Mann-Whitney U test	Effect Size
Cereals and Grains (g/1000kcal)	M	21 (70)	139.9	127.0	159.3	140.5 ± 8.8	108	+30	U = 65, p = 0.19	r=0.24
	F	9 (30)	145.2	130.5	165.1	146.1 ± 11.1	108	+35		
	T	30 (100)	140.6	127.0	165.1	142.2 ± 9.7	108	+31		
Pulses and legumes (g/1000kcal)	M	21 (70)	11.7	10.5	53.1	15.5 ± 11.0	11	+40.9	t(28) = -0.16, p = 0.87	d=0.06
	F	9 (30)	13.2	11.7	26.9	16.1± 5.80	11	+46.3		
	T	30 (100)	11.9	10.5	53.1	15.7 ± 9.60	11	+42.7		
Green Leafy vegetables (g/1000kcal)	M	21 (70)	18.7	0	46.5	15.1 ± 12.8	40	-62	t(28) = 0.65, p = 0.52	d=0.26
	F	9 (30)	19.6	0	22.4	11.9 ± 11.3	40	-70		
	T	30 (100)	18.7	0	46.5	14.1 ± 12.3	40	-65		
Other vegetables (g/1000kcal)	M	21 (70)	77.0	39.8	93.0	74.7 ± 11.6	54	+38	U = 88, p = 0.78	r=0.05
	F	9 (30)	74.3	43.5	92.8	73.5 ± 16.1	54	+36		
	T	30 (100)	76.8	39.8	93.0	74.4 ± 12.8	54	+38		
Roots and Tubers (g/1000 kcal)	M	21 (70)	48.7	0	57.5	39.7 ± 19.3	41	-2.5	U = 88, p = 0.7	r=0.15
	F	9 (30)	44.0	0	64.4	36.6 ± 24.2	41	-11		
	T	30 (100)	47.6	0	64.4	38.8 ± 20.5	41	-5		
Fruits (g/1000 kcal)	M	21 (70)	75	0	93.0	66.6 ± 24.6	40	+66	U = 89, p = 0.82	r=0.05
	F	9 (30)	78.4	0	89.8	52.2 ± 41.7	40	+30		
	T	30 (100)	75	0	93.0	62.3 ± 30.7	40	+55		
Processed fruit products (g/1000 kcal)	M	21 (70)	7.6	0	9.6	5.9 ± 3.9	5	+18	t(28) = -0.03, p = 0.97	d=0.01
	F	9 (30)	8.5	0	9.9	5.9 ± 4.4	5	+18		
	T	30 (100)	7.7	0	9.9	5.9 ± 3.7	5	+18		
Eggs (g/1000 kcal)	M	21 (70)	57.5	0	65.1	48.8 ± 18.3	27	+80	U = 73, p = 0.34	r=0.18
	F	9 (30)	43.5	0	69.6	39.3 ± 24.7	27	+46		
	T	30 (100)	54.8	0	69.6	45.9 ± 20.5	27	+70		
Poultry (g/1000 kcal)	M	21 (70)	58.0	0	93.0	53.49 ± 21.2	67.5	-21	t(10.9)=0.2, p = 0.77	d=0.1
	F	9 (30)	66.0	0	83.9	50.06 ± 32.8	67.5	-24		
	T	30 (100)	58.6	0	93.0	52.46 ± 24.6	67.5	-23		
Milk and milk products (ml/1000 kcal)	M	21 (70)	250	0	303.0	140.4 ± 74.3	203	-31	U = 89, p = 0.82	r=0.05
	F	9 (30)	250	0	220.0	119.9 ± 84.9	203	-41.4		
	T	30 (100)	108.6	0	303.0	133.7 ± 75.9	203	-34.2		
Added sugar (g/1000 kcal)	M	21 (70)	15.8	14.2	21.0	16.4 ± 1.7	16	+2	U = 72, p = 0.32	r=0.19
	F	9 (30)	17.0	13.9	19.8	16.8 ± 1.7	16	+5		
	T	30 (100)	15.6	13.9	21.0	16.6 ± 2.5	16	+3		
Cooking oil (ml/1000 kcal)	M	21 (70)	15.4	11.2	18.6	15.5 ± 1.7	14	+10	U = 43, p = 0.02	r=0.43
	F	9 (30)	17.6	13.0	23.2	17.5 ± 2.6	14	+25		
	T	30 (100)	15.7	11.2	23.2	16.1 ± 2.2	14	+15		
Solid fats (g/1000 kcal)	M	21 (70)	0	0	9.9	1.0 ± 2.4	5	-80	t(28) = 0.1, p = 0.91	d=0.04
	F	9 (30)	0	0	4.2	0.9 ± 1.7	5	-82		
	T	30 (100)	0	0	9.9	0.9 ± 2.2	5	-82		

Consumption of packaged processed foods

The consumption frequency of packaged processed foods is presented in Table 4. Aerated/soft/cold drinks and diet beverages were avoided by all participants. Fruit juices were consumed by 53% of participants on a monthly basis and 47% consumed them rarely. Flavoured milk was consumed frequently by 13.3% of participants on a fortnightly basis, 16.7% of participants had it on a monthly basis, and 70% of participants had it on a yearly basis. Yogurt/curd-based drinks were also popular, with 20% consuming them weekly, 23.2% consuming them fortnightly, and 30% consuming them monthly. Ready-to-drink coffee/tea mixes, vegetable juice, and plant milk substitutes were never consumed by any participants. Bread and bakery products were consumed frequently, with varying frequencies across different intervals, suggesting their importance in the daily diet. Indian sweets were fortnightly indulgence for 73.3% of participants, while ice cream/kulfi and chocolate bars were had fortnightly by 63.3% and 60% of participants, respectively. Packaged condiments such as sauces, chutneys, and pickles were commonly consumed, primarily on a daily or alternate-day basis, highlighting their significance in Indian cuisine. Ready-to-cook (RTC) snacks, both vegetarian and non-vegetarian, were not popular among the participants. Moreover, mixes of bread and dessert were consumed yearly by 90% and 66.7% of participants, respectively.

**Table 4:** Food frequency of packaged processed foods n: Number, %: Percentage

Food Products	Daily n (%)	Alternate Days n (%)	Twice a Week n (%)	Weekly n (%)	Fortnightly n (%)	Monthly n (%)	Yearly n (%)	Rarely/Never n (%)
Aerated/soft/cold drinks	-	-	-	-	-	-	30 (100)	-
Diet beverages	-	-	-	-	-	-	30 (100)	-
Fruit juices	-	-	-	-	-	16 (53)	14 (47)	-
Vegetable juices	-	-	-	-	-	-	30 (100)	-
Flavoured milk	-	-	-	-	4 (13.3)	5 (16.7)	21 (70)	-
Yogurt/curd-based drinks	-	-	-	6 (20)	7 (23.2)	9 (30)	4 (13.4)	4 (13.4)
Plant-based milk substitutes	-	-	-	-	-	-	-	30 (100)
Ready-to-drink coffee/tea mixes	-	-	-	-	-	-	-	30 (100)
Bread	5 (16.7)	15 (50)	8 (26.6)	2 (6.7)	-	-	-	-
Biscuits/cookies/cream rolls	7 (23.3)	3 (10)	5 (16.7)	5 (16.7)	4 (13.3)	6 (20)	-	-
Namkeen	-	-	5 (16.7)	16 (53.3)	9 (30)	-	-	-
Chips	-	-	6 (20)	5 (16.7)	9 (30)	3 (10)	7 (23.3)	-
Breakfast cereals	2 (6.7)	4 (13.3)	2 (6.7)	2 (6.7)	1 (3.3)	9 (30)	8 (26.6)	2 (6.7)
Bakery products	-	-	-	1 (3.3)	4 (13.3)	20 (66.7)	5 (16.7)	-
Indian sweets	-	-	-	-	22 (73.3)	6 (20)	2 (6.7)	-
Flavoured yoghurt/kheer/payasam	-	1 (3.3)	4 (13.3)	16 (53.3)	5 (16.7)	2 (6.7)	2 (6.7)	-
Fruit yoghurt	-	-	-	3 (10)	19 (63.3)	8 (26.7)	-	-
Ice cream/kulfi	-	-	-	3 (10)	19 (63.3)	8 (26.7)	-	-
Fruit jam	3 (10)	15 (50)	4 (13)	5 (17)	3 (10)	-	-	-
Toffee/lollipops/fruit candies	-	-	-	-	8 (26.6)	22 (73.4)	-	-
Chocolate bar	-	-	-	-	18 (60)	9 (30)	3 (10)	-
Sauces	14 (46.7)	7 (23.3)	1 (3.3)	1 (3.3)	2 (6.7)	1 (3.3)	1 (3.3)	3 (10)
Chutney	14 (46.7)	7 (23.3)	1 (3.3)	1 (3.3)	2 (6.7)	1 (3.3)	1 (3.3)	3 (10)
Pickle	6 (20)	6 (20)	8 (26.6)	5 (16.6)	1 (3.3)	1 (3.3)	1 (3.3)	2 (6.6)
Gravies/curries/pastes	-	-	-	10 (33.3)	10 (33.3)	5 (16.7)	5 (16.7)	-
Cheese/milk-based spreads	-	-	-	-	15 (50)	10 (33.3)	3 (10)	2 (6.7)
Nut butter/spreads	8 (26.7)	4 (13.3)	2 (6.7)	6 (20)	2 (6.7)	3 (10)	2 (6.7)	3 (10)
Butter/flavoured butter	-	2 (6.7)	-	4 (13.3)	19 (63.3)	3 (10)	2 (6.7)	-
Instant soup/pasta/noodles/oats/upma	-	-	-	-	23 (76.7)	4 (13.3)	3 (10)	-
RTC snack mixes	-	-	-	-	-	12 (40)	15 (50)	3 (10)
RTC veg snacks	-	-	-	-	-	-	-	30 (100)
Frozen paratha/spring roll sheets	-	-	-	-	-	-	-	30 (100)
RTC non-veg snacks	-	-	-	-	-	2 (6.7)	7 (23.3)	21 (70)
RTC meal in cups	-	-	-	-	-	-	-	30 (100)
Bread mixes	-	-	-	-	-	-	27 (90)	3 (10)
Dessert mixes	-	-	-	-	-	8 (26.7)	20 (66.7)	2 (6.7)

Consumption of sports foods and sports supplements

Around 56.7% of the participants consumed protein powder daily, 16.6% rarely or yearly, and 26.7% never. Around 76.6% of the participants reported yearly consumption, 16.7% opted for it once a month, while 6.7% rarely had protein bars. Around 36.7% had energy bars rarely, 20% never consumed energy bars, 6.7% had them fortnightly, and 6.6% had them monthly. Around 10% of boxers consumed isotonic sports drinks daily, 6.7% on alternate days, and 20% on a yearly basis. Around 46.7% of the participants had electrolyte drinks on a monthly basis, and 36.7% had it rarely.

Consumption of dietary supplements

Around 16.7% of the boxers daily consumed multivitamin-multimineral supplements, while 83.3% had it rarely. Around 3.3% incorporated calcium supplements daily, while 96.7% never consumed them. None of the athletes consumed iron-folic acid (IFA) tablets in the present study. Around 16.6% of participants had vitamin D supplements once a week in the form of powder.

Nutrient intake

Descriptive statistics such as mean, median, standard deviation, and range of intake of nutrient intake by boxers and inferential statistics such as the Mann-Whitney U test and effect size for gender-wise differences in intake of nutrients are presented in Table 5. The mean intake of energy, carbohydrate, protein, dietary fat, and dietary fibre was 2475±209 kcal/day, 304.7±26.7 g/day, 93±18.3 g/day, 95.8±9.2 g/day, and 40.9±3.7 g/day, respectively. There was a significant difference with a large effect size between male and female boxers for energy intake (U=22, r=0.6). There was a highly significant difference with a large effect size between male and female boxers for carbohydrate intake (U=22, r=0.59), protein intake (U=24, r=0.58), and dietary fibre intake (U=24, r=0.58). The gender-wise difference in intake of dietary fat was marginally significant with a medium effect size (U=51, p=0.053, r=0.36).

**Table 5:** Energy (kcal/day), macronutrients (g/day), and dietary fibre (g/day) intake according to gender G: Gender, n: Frequency, %: Percentage, Mdn.: Median, Min.: Minimum, Max.: Maximum, S.D.: Standard Deviation, p≤0.05: Significant, p≤0.01: Highly Significant

	Descriptive Statistics							Inferential Statistics	
	G	n (%)	Mdn.	Min.		Max.	Mean ± S.D.	Mann-Whitney U test	Effect Size (r)
Energy (kcal/day)	M	21 (70)	2583		2150	2834	2559 ± 173	U=22, p=0.001	0.6
	F	9 (30)	2272		2017	2548	2279 ± 148		
	T	30 (100)	2521		2017	2834	2475 ± 209		
Carbohydrates (g/day)	M	21 (70)	317.3		268.9	346.1	315.1 ± 18.4	U=23, p=0.001	0.59
	F	9 (30)	287.9		236.9	313.6	280.6 ± 28.4		
	T	30 (100)	311.4		236.9	346.1	304.7 ± 26.7		
Protein (g/day)	M	21 (70)	105.3		45.8	121.5	99.3 ± 17.7	U=24, p=0.001	0.58
	F	9 (30)	77.6		65.0	94.7	78.3 ± 9.2		
	T	30 (100)	97.3		45.8	121.5	93.0 ± 18.3		
Dietary fat (g/day)	M	21 (70)	98.3		72.7	118.7	97.7 ± 9.5	U=51, p=0.053	0.36
	F	9 (30)	88.9		80.1	101.7	91.5 ± 7.3		
	T	30 (100)	96.8		72.7	118.7	95.8 ± 9.2		
Dietary fibre (g/day)	M	21 (70)	45.5		41.8	51.8	46.0 ± 3.0	U=24, p=0.002	0.58
	F	9 (30)	41.3		34.6	45.1	40.9 ± 3.7		
	T	30 (100)	44.8		44.8	51.8	44.5 ± 4.0		

Energy and macronutrient recommendations given in the Nutrition and Hydration Guidelines for Excellence in Sports Performance were utilized to assess the adequacy of nutrients [1]. Descriptive statistics such as mean, median, standard deviation, and range of intake of nutrients per kg body weight per day by boxers and inferential statistics such as the t-test/Mann-Whitney U test and effect size for gender-wise differences in intake of energy and macronutrients are presented in Table 6. The mean intake of energy, carbohydrate, protein, and dietary fat was 43±3.4 kcal/kg/day, 5.3±0.4 g/kg/day, 1.6±0.3 g/kg/day, and 1.6±0.1 g/kg/day, respectively. There was a significant difference with a large effect size between male and female boxers for energy intake (U=25, r=0.52), carbohydrate intake (U=34, r=0.50), and protein intake (U=29, r=0.54). The gender-wise difference in intake of dietary fat was non-significant.

**Table 6:** Energy (kcal/kg/day) and macronutrients (g/kg/day) intake according to gender G: Gender, n: Frequency, %: Percentage, Mdn.: Median, Min.: Minimum, Max.: Maximum, S.D.: Standard Deviation, p≤0.05: Significant, p≤0.01: Highly Significant

	Descriptive Statistics								Inferential Statistics	
	G	n (%)	Mdn.	Min.	Max.	Mean ± S.D.	Recommendation	% of recommendations fulfilled	Mann-Whitney U test	Effect Size (r)
Energy (kcal/kg/day)	M	21 (70)	44	37	49	44 ± 2.8	60	-27	U=25, p=0.005	0.52
	F	9 (30)	41	34	44	40 ± 3.2	60	-33		
	T	30 (100)	44	34	49	43 ± 3.4	60	-29		
Carbohydrates (g/kg/day)	M	21 (70)	5.4	4.6	6.1	5.4 ± 0.2	8.3	-33.9	U=34, p=0.007	0.50
	F	9 (30)	5.2	4.0	5.5	5.0 ± 0.5	8.3	-39.8		
	T	30 (100)	5.4	4.0	6.1	5.3 ± 0.4	8.3	-36.2		
Proteins (g/kg/day)	M	21 (70)	1.8	0.7	2.0	1.7 ± 0.3	2.5	-32.0	U=29, p=0.003	0.54
	F	9 (30)	1.4	1.1	1.7	1.4 ± 0.2	2.5	-44.0		
	T	30 (100)	1.7	0.7	2.0	1.6 ± 0.3	2.5	-34.8		
Dietary fat (g/kg/day)	M	21 (70)	1.7	1.3	2.0	1.7 ± 0.1	2.1	-10.1	U=66, p=0.20	0.24
	F	9 (30)	1.6	1.4	1.9	1.6 ± 0.1	2.1	-23.9		
	T	30 (100)	1.7	1.3	2.0	1.6 ± 0.1	2.1	-23.9		

Descriptive statistics such as mean, median, standard deviation, range of intake, percentage of EAR of micronutrients fulfilled by boxers, and inferential statistics such as the t-test/Mann-Whitney U test and effect size for gender-wise differences in intake of micronutrients are presented in Table 7. The mean intake of calcium, iron, and zinc was 827.0±300.5 mg/day, 16.4±1.8 mg/day, and 10.5±1.1 mg/day, respectively. The mean folate intake was 185.3±31.4 ug/day. Females could not fulfill EAR for calcium and iron. Both genders could not fulfill zinc and folate recommendations (EAR). The mean vitamin C intake was 107.9±15.3 mg/day, and both genders exceeded the recommendation (EAR). The gender-wise difference in intake of calcium and vitamin C was statistically insignificant, while the difference was statistically significant with a large effect size for iron intake (U=22.5, r=0.59), zinc intake (U=23.5, r=0.59), and folate intake (U=30, r=0.53).

**Table 7:** Intake of micronutrients according to gender G: Gender, n: Frequency, %: Percentage, Mdn.: Median, Min.: Minimum, Max.: Maximum, S.D.: Standard Deviation, p≤0.05: Significant, p≤0.01: Highly Significant

	Descriptive Statistics								Inferential Statistics	
	G	n (%)	Mdn.	Min.	Max.	Mean ± S.D.	Recommendation (EAR)	% of recommendations fulfilled	Mann-Whitney U test	Effect Size (r)
Calcium (mg/day)	M	21 (70)	779.7	281.2	1507.5	883.3 ± 290.0	800	+10.4	U = 59, p = 0.116	0.29
	F	9 (30)	715.9	239.2	1033.2	697.7 ± 300.0	800	-12.8		
	T	30 (100)	771.6	239.2	1507.5	827.0 ± 300.5	800	+3		
Iron (mg/day)	M	21 (70)	17.3	13.9	19.2	17.2 ± 1.4	11	+56.3	U = 22.5, p = 0.001	0.59
	F	9 (30)	14.3	12.7	17.4	14.8± 1.6	15	-1.4		
	T	30 (100)	16.7	12.7	19.2	16.4 ± 1.8				
Zinc (mg/day)	M	21 (70)	11.1	8.4	12.3	10.9 ± 0.8	14	- 22.2	U = 23.5, p = 0.002	0.59
	F	9 (30)	9.3	8.07	11.2	9.5 ± 0.9	11	- 13.7		
	T	30 (100)	10.8	8.07	12.3	10.5 ± 1.1				
Folate (ug/day)	M	21 (70)	194.4	153.3	264.3	196.3 ± 27.8	250	-21	U = 30, p = 0.004	0.53
	F	9 (30)	159.5	114.8	190.6	159.4 ± 24.0	180	-11.5		
	T	30 (100)	183.8	114.8	264.3	185.3 ± 31.4				
Vitamin C (mg/day)	M	21 (70)	108.9	89.2	138.1	110.5 ± 15.0	65	+69	U = 67, p = 0.22	0.23
	F	9 (30)	104.8	78.5	117.6	101.7 ± 14.8	55	+83		
	T	30 (100)	108.2	78.5	138.1	107.9 ± 15.3				

Anthropometric measurements and body composition

Table 8 presents descriptive statistics of anthropometric measurements and body composition gender-wise along with inferential statistics such as t-tests and effect size for gender-wise differences. The mean body weight (kg) of boxers was 55.6±7.51. The mean body weight (kg) of male and female boxers was 55±2.21 and 49±2.06, respectively. The mean body height (m) of boxers was 1.68±0.08. The mean body height (m) of male and female boxers was 1.71±0.06 and 1.56±0.06, respectively. The mean BMI (kg/m^2^) of boxers was 20.33±1.86. The mean BMI (kg/m^2^) of male and female boxers was 19.57±1.58 and 22.1±1.13, respectively. Among boxers, 36.67% (n=11) of participants were underweight, 46.67% (n=14) had a normal BMI, and 16.67% (n=5) were overweight. The mean body fat (%) for boxers was 13.11±1.8. The mean body fat (%) of male and female boxers was 12.69±1.82 and 13.29±1.8, respectively. The mean body visceral fat (%) of boxers was 2.73±0.87. The mean body visceral fat (%) of male and female boxers was 2.57±0.81 and 3.11±0.93, respectively. The mean body muscle (%) of boxers was 35.57±2.63. The mean body muscle (%) of male and female boxers was 37±1.29 and 32.22±1.72, respectively. The mean BMI (kg/m^2^) and body muscle (%) were significantly different gender-wise and had a large effect size.

**Table 8:** Anthropometric measurements and body composition according to gender G: Gender, n: Frequency, %: Percentage, Mdn.: Median, Min.: Minimum, Max.: Maximum, S.D.: Standard Deviation, p≤0.05: Significant, p≤0.01: Highly Significant

Gender	n (%)	Body Height (m)	Body Weight (kg)	BMI (kg/m^2^)	Body Fat %	Visceral Fat %	Body Muscle %
Total	30 (100)	1.68 ± 0.08	55.6 ± 7.51	20.33 ± 1.86	13.11 ± 1.8	2.73 ± 0.87	35.57 ± 2.63
Male	21 (70)	1.72 ± 0.05	58.43 ± 7.22	19.57 ± 1.58	12.8 ± 1.85	2.57 ± 0.81	37 ± 1.29
Female	9 (30)	1.59 ± 0.06	49 ± 2.06	22.1 ± 1.13	13.82 ± 1.53	3.11 ± 0.93	32.22 ± 1.72
t-test		t(28) = 5.42	t(25.99) = 5.49	t(28) = -4.33	t(28) = -1.45	t(28) = -1.6	t(12.08) = 7.5
p-value		p = 0.001	p = 0.001	p = 0.001	p =0.157	p = 0.12	p = 0.001
Effect size		d =2.16 (large effect)	d =2.19 (large effect)	d=1.73 (large effect)	d =0.58 (medium effect)	d=0.64 (medium effect)	d=2.99 (large effect)

Exercise energy expenditure

The mean exercise energy expenditure was 930±65.13 kcal/day.

Energy availability

The mean EA of boxers was 29.28±3.79 kcal/kg FFM/day. Among male boxers, the mean EA was 29.71±3.74 kcal/kg FFM/day and a median EA of 29.6 kcal/kg FFM/day, spanning from 20.74 to 36.6 kcal/kg FFM/day. Among female boxers, the mean EA was 28.3±3.95 kcal/kg FFM/day, and the median EA was 26.87 kcal/kg FFM/day, ranging from 23.47 to 36.17 kcal/kg FFM/day. Around 66.67% of boxers in the present study had LEA while the rest had reduced EA.

As shown in Table 9, there was a positive correlation between EA (kcal/kg FFM/day) and intake of cereals and grains(r=0.2), pulses and legumes (r=0.22), green leafy vegetables (r=0.26), other vegetables (r=0.31), roots and tubers (r=0.16), fruits (r=0.16), nuts (r=0.1), cooking oil (0.01), solid fats (r=0.1), added sugar(r=0.14), and nuts and seeds (r=0.1), but the correlation was not statistically significant.

**Table 9:** Correlation between energy availability (kcal/kg FFM/day) and food intake

	r-value	p-value
Energy availability (kcal/kg FFM/day)	1	
Cereals and grains (g/day)	0.20	0.291
Pulses and legumes (g/day)	0.22	0.248
Poultry (g/day)	-0.03	0.859
Eggs (g/day)	-0.20	0.277
Green leafy vegetables (g/day)	0.26	0.163
Other vegetables (g/day)	0.31	0.097
Roots and tubers (g/day)	0.16	0.395
Fruits (g/day)	0.16	0.412
Milk and milk products (mL/day)	0	0.982
Cooking oil (mL/day)	0.01	0.977
Solid fats (g/day)	0.10	0.585
Added sugar (g/day)	0.14	0.471
Nuts and seeds (g/day)	0.10	0.598

As shown in Table 10, EA (kcal/kg FFM/day) positively correlated with energy (kcal/day) and macronutrient (g/day) intake. This shows that the higher the energy (kcal/day) and macronutrient (g/day) intake, the higher the EA (kcal/kg FFM/day) of the participants. The highest correlation was found between EA (kcal/kg FFM/day) and total energy intake per day (kcal/day).

**Table 10:** Correlation of energy availability (kcal/kg FFM/day) with energy and macronutrient intake

	r-value	p-value
Energy Availability (kcal/kg FFM/day)	1	
Energy (kcal/day)	0.59	0.001
Energy (kcal/ kg/day)	0.53	0.002
Carbohydrates (g/day)	0.38	0.038
Carbohydrates (g/kg/day)	0.33	0.076
Protein (g/day)	0.47	0.008
Protein (g/kg/day)	0.44	0.016
Fat (g/day)	0.55	0.002
Fat (g/kg/day)	0.47	0.008

As shown in Table 11, EA (kcal/kg FFM/day) was positively correlated and highly significant with the total daily intake of iron (r=0.43), zinc (r=0.44), phosphorus (r=0.50), potassium (r=0.54), selenium (r=0.46), niacin (r=0.53), riboflavin (r=0.47), folate (r=0.43), and vitamin C (r=0.46). EA (kcal/kg FFM/day) was positively correlated and significant with the intake of biotin (r=0.36), while EA (kcal/kg FFM/day) positively correlated with calcium intake (r=0.07), but it was not statistically significant.

**Table 11:** Correlation between energy availability (kcal/kg FFM/day) and micronutrient intake

	r-value	p-value
Energy Availability (kcal/kg FFM/day)	1	
Calcium (mg/day)	0.07	0.712
Phosphorus (mg/day)	0.5	0.005
Iron (mg/day)	0.43	0.018
Potassium (mg/day)	0.54	0.002
Zinc (mg/day)	0.44	0.014
Selenium (ug/day)	0.46	0.01
Vitamin C (mg/day)	0.46	0.01
Riboflavin (mg/day)	0.47	0.008
Niacin (mg/day)	0.53	0.003
Biotin (mg/day)	0.36	0.048
Folate (ug/day)	0.43	0.019

As shown in Table 12, EA (kcal/kg FFM/day) correlated positively and highly significantly with the Omega 3 intake (r=0.47). EA (kcal/kg FFM/day) positively correlated with total daily intake of monounsaturated fatty acids (MUFA), polyunsaturated fatty acids (PUFA), and total saturated fatty acids (TSFA) but was not statistically significant.

**Table 12:** Correlation between energy availability (kcal/kg FFM/day) and fatty acid intake MUFA: Monounsaturated Fatty Acids, PUFA: Polyunsaturated Fatty Acids, TSFA: Total Saturated Fatty Acids

		Omega 3 (mg)	MUFA (g)	PUFA (g)	TSFA (g)
Energy availability (kcal/kg FFM/day)	r-value	0.47	0.17	0.36	0.33
	p-value	0.009	0.37	0.051	0.077

As shown in Table 13, EA had a moderately significant inverse relationship with fat-free mass (%). The higher the fat-free mass among participants, the lower the EA in the present study. The basal metabolic rate is estimated by fat-free mass since fat-free mass is the actively metabolizing tissue [12]. The larger a participant's fat-free mass, the greater the rate of energy expenditure, which reduces EA. Hence, it is important to fulfill total energy requirements to maintain optimal EA as per the energy expended during exercise [13].

**Table 13:** Correlation of energy availability (kcal/kg FFM/day) with fat-free mass (%) and exercise energy expenditure (kcal)

		Fat-Free Mass (%)	EEE (kcal)
Energy availability (kcal/kg FFM/day)	r-value	-0.54	-0.08
	p-value	0.002	0.674

As shown in Table 14, the influence of each parameter such as FFM (%), EEE (kcal), and energy intake (kcal) on the EA (kcal/kg FFM/day) was highly significant using a multiple linear regression analysis. The regression model showed that the variables FFM (%), EEE (kcal), and energy intake (kcal) explained 99.03% of the variance from the variable EA (kcal/kg FFM/day). An ANOVA was used to test whether this value was significantly different from zero. It was found that the effect was significantly different from zero, F=880.63, p=0.001, and R2=0.99. The following regression model is obtained: EA = 27.36 - 0.57 · FFM % - 0.02 · EEE + 0.02 · Energy.

**Table 14:** Influence of FFM (%), EEE (kcal), and energy intake (kcal) on the energy availability (kcal/kg FFM/day)

	Unstandardized Coefficients	Standardized Coefficients			
Model	B	Beta	Standard error	t	p-value
Constant	27.36		1.66	16.44	0.001
Fat free mass(%)	-0.57	-0.84	0.01	-40.75	0.001
EEE (kcal)	-0.02	-0.25	0	-12.93	0.001
Energy (kcal)	0.02	0.89	0	42.81	0.001

As shown in Table 15, energy intake (kcal) was significantly related to EA (kcal/kg FFM/day) and total body muscle (%) moderated that relationship (B=0.01, SE=0, p=0.004). Similarly, in Table 16, energy (kcal)/kg/day was significantly related to EA (kcal/kg FFM/day) and total body muscle (%) moderated that relationship (B=0.32, SE=0.1, p=0.004).

**Table 15:** Energy availability (kcal/kg FFM/day) predicted by energy intake (kcal) and total body muscle percentage

	Unstandardized Coefficients		Standardized Coefficients			
Predictor	B	Beta		Standard error	t	p-value
Constant	-3.96			8.91	-0.44	0.661
Energy (kcal) (X)	0.01	0.62		0	3.88	0.001
Total body muscle (%) (W')	-12.71	-7.08		3.94	-3.22	0.003
(X * W')	0.01	6.92		0	3.13	0.004

**Table 16:** Energy availability (kcal/kg FFM/day) predicted by energy/kg/day and total body muscle percentage

	Unstandardized Coefficients	Standardized Coefficients			
Predictor	B	Beta	Standard error	t	p-value
Constant	-2.69		9.42	-0.29	0.778
Energy/kg/day (kcal/kg/day) (X)	0.78	0.56	0.22	3.59	0.001
Total body muscle(%) (W')	-14.02	-7.82	4.44	-3.16	0.004
(X * W')	0.32	7.74	0.1	3.13	0.004

## Discussion

Boxing is an extreme contact sport [14]. In boxing, athletes are categorized based on their body mass to minimize differences between competitors. Boxers often decrease their body mass to compete in a lower-weight class. Acute weight reduction should not exceed 5% of body mass as it would increase the risk of cardiovascular and endocrine disorders or even death. Gradual fat reduction using optimal nutrition and supplement strategies can enhance sports performance and improve body composition [15].

Body composition is crucial for boxers as it affects their performance in the ring. Boxers may get an advantage with higher muscle mass. This is because muscle mass generates more force and power, which is important for sports-specific movements such as punching or grappling with an opponent. Muscle mass generates power, whereas excess body fat acts as a dead weight that must be repeatedly lifted against gravity reducing performance in the ring [16]. Appropriate nutrition and training strategies aid in improving the body composition of combat sports athletes. The primary source of energy for the boxers is carbohydrates to ensure the proper level of muscle glycogen, which is essential for intense and long hours of training [15]. Intake of high biological value protein sources is required for muscle protein synthesis, while essential fats are crucial for recovery [15]. Timing of nutrient intake is also an important topic in sports nutrition. If the energy intake does not fulfill the energy expended by athletes during training, it may increase muscle breakdown, compromise immune function, cause delays in injury recovery, and increase the risk of LEA. Dietary restrictions have been associated with decreased power-related performance outcomes among male combat athletes [17].

Micronutrient deficiencies in athletes negatively affect health and sports performance [18]. Strenuous and long hours of training with dietary restrictions may cause the wasting of muscles, which increases the risk of LEA. Previous studies reported female athletes exhibited a reduction in reproductive hormones after four days of LEA, a decrease in blood glucose levels, suppression of blood insulin and blood leptin, and an increase in blood cortisol after five days of LEA [19,20]. Additionally, research has demonstrated that female athletes are more susceptible to stress fractures and osteoporosis due to a higher rate of bone resorption than their male counterparts [21,22]. A reduction in coordination and concentration during training was self-reported by athletes with a high risk of LEA [23]. At present, there is no Indian study that has evaluated the EA of adult Indian boxers and examined the factors that contribute to LEA. This study was undertaken with the objectives of assessing the EA of Indian boxers (lightweight category) and correlating it with contributing factors such as anthropometric measurements, body composition, dietary intake, nutrient intake, and exercise energy expenditure.

The average BMI (kg/m^2^) of boxers in this study was 20.33±1.86, which is similar to the BMI of adolescent Indian elite boxers (20.9±2.4) and Lithuanian boxers (20.7±3.2) [24,25]. The average BMI (kg/m^2^) of male boxers in this study was lower (19.57±1.58) compared to elite Olympic male boxers (22.7±2.1). However, the average BMI (kg/m^2^) of female boxers in our study (22.1 ± 1.13) was similar to that of elite Olympic female boxers (22.5±3.2) [26]. The mean BMI (kg/m^2^) was significantly different gender-wise and had a large effect size in the present study, but the gender-wise difference was insignificant in the previous study [24]. The mean body fat (%) for boxers (13.11±1.8) in the present study was similar to adolescent Indian elite boxers (13.1±2.8) and Lithuanian boxers (13.4 ± 6.4) but lower than the Polish boxers (15.5±4.7) [24,25,27].

The average energy intake (kcal/kg/day) was 43±3.4 in the present study, which was lower than that of elite Indian boxers (45.5) and high-performance combat athletes from Lithuania (51.4±17) [28,25]. Along with energy intake, exercise energy expenditure in the present study was also lower than reported in the previous study [24]. The mean carbohydrate intake (g/kg/d) in the current investigation was 5.3±0.4 g/kg/day, which was lower than that of the previous study among boxers from India (6.2) and Lithuania (6.3) [28,25]. However, the mean protein intake (g/kg/ day) was similar among boxers from the present study (1.6±0.3) and that of Indian elite boxers and high-performance combat athletes from Lithuania (1.7±0.6) [28,25]. The mean dietary fat intake (g/day) of boxers was 95.8±9.2 in the present study and was higher than reported by the previous investigator (77.1±22.8) [24]. There were significant differences in the intake of cereals and grains, other vegetables, and added sugar based on gender. Around 29% of the recommended energy/kg/day intake was not fulfilled. Recommended carbohydrate/kg/day, protein/kg/day, and fat/kg/day intake were not fulfilled by 36.2%, 34.8%, and 23.9%, respectively. Repeated exposures to low carbohydrate intake (LCHO) along with LEA may impair exercise capacity, recovery, training adaptations, and other performance outcomes [29]. Low carbohydrate intakes increase the risk of RED-S in female athletes [29]. Athletes failed to meet the recommended intake for the food groups, energy intake, and macronutrient intake in the present study. Females could not meet the EAR for calcium and iron. Both genders could not meet zinc and folate recommendations (EAR). Extensive studies on nutrient and food intake before and after energy adjustments of Indian boxers were not done previously.

The mean EA of boxers was 29.28±3.79 kcal/kg FFM/day in the present study with little or no gender differences (29.71±3.74 kcal/kg FFM/day in males versus 28.3±3.95 kcal/kg FFM/day in females). A previous study reported close enough EA (27.8±9.8 kcal/kg FFM/day) for male boxers, while much lower EA for female boxers as compared to the present study (19.5±5.6 kcal/kg FFM/day) [24]. Around 66.67% of boxers in the present study had low energy availability, while the rest had reduced energy availability. A higher prevalence (87.5%) of LEA (21.5±6.1 kcal/kg FFM) has been reported among adolescent Indian elite boxers by another investigator [24]. There was no significant correlation between various food groups and EA in the present study. EA was positively correlated with energy, macronutrient, and micronutrient (iron, zinc, phosphorus, potassium, selenium, niacin, riboflavin, folate, and vitamin C) intake. The multiple linear regression analysis demonstrates that FFM (%), EEE (kcal), and energy intake (kcal) were highly significant predictors of EA (kcal/kg FFM/day) in the present study.

Limitations

In the present study, EA was monitored using estimations of mean energy intake from three-day dietary recall, and mean exercise energy expenditure was calculated using three-day training records. Thus, relying heavily on self-reporting of the athlete. There can be under-reporting and over-reporting of energy intake and energy expenditure. Further, these estimations do not capture cumulative energy availability, including the variation in energy intake and training across a week. No biochemical tests were assessed. Bone mineral density was also not assessed. Eating disorders among athletes can negatively affect EA. Eating disorders were not evaluated.

## Conclusions

The majority of the boxers had normal BMI. The mean body fat and visceral fat were higher in female boxers than in males. The lower the body fat (%) of boxers, the higher the energy intake and energy/kg of body weight/day. The mean body muscle was higher in males than in female boxers. Participants did not have adequate intake of various food groups before and after adjustment of the energy intake. Processed packaged food consumption was popular among the participants. Sports foods and supplements were consumed by a few athletes. Dietary supplements were not common among participants. The nutrient intake of the participants was inadequate. The majority of the boxers could not fulfill energy/kg/day, carbohydrate/kg/day, protein/kg/day, and fat/kg/day requirements. Iron and calcium intake was inadequate among female participants.

The majority of athletes had low energy availability. The higher the energy and nutrient intake, the higher the energy availability of the participants. Energy intake was significantly related to energy availability and total body muscle % significantly moderated that relationship. The influence of FFM (%), EEE (kcal), and energy intake (kcal) on energy availability (kcal/kg FFM/day) was significant using multiple linear regression analysis. The nutrition of athletes needs to be optimized, individualized, and adjusted to meet their daily energy requirements and achieve body composition goals as per the training periodization and competition season. Hence, regular dietary assessment and nutrition counseling are required to address the issues of low energy availability and make healthy dietary choices.

## References

[1] (2024). Nutrition and hydration guidelines for excellence in sports performance. http://ilsi-india.org/PDF/Nutrition_&_Hyd_Guidelines_for_Athletes_Final_report.pdf.

[2] Mountjoy M, Sundgot-Borgen JK, Burke LM (2018). IOC consensus statement on relative energy deficiency in sport (RED-S): 2018 update. Br J Sports Med.

[3] Areta JL, Taylor HL, Koehler K (2021). Low energy availability: history, definition and evidence of its endocrine, metabolic and physiological effects in prospective studies in females and males. Eur J Appl Physiol.

[4] Logue D, Madigan SM, Delahunt E, Heinen M, Mc Donnell SJ, Corish CA (2018). Low energy availability in athletes: a review of prevalence, dietary patterns, physiological health, and sports performance. Sports Med.

[5] Logue DM, Madigan SM, Melin A, Delahunt E, Heinen M, Donnell SM, Corish CA (2020). Low energy availability in athletes 2020: an updated narrative review of prevalence, risk, within-day energy balance, knowledge, and impact on sports performance. Nutrients.

[6] Mountjoy M, Sundgot-Borgen J, Burke L (2014). The IOC consensus statement: beyond the Female Athlete Triad--Relative Energy Deficiency in Sport (RED-S). Br J Sports Med.

[7] Taguchi M, Manore MM (2022). Reexamining the calculations of exercise energy expenditure in the energy availability equation of free-living athletes. Front Sports Act Living.

[8] (2024). Sports Dietitians Australia (SDA): Boxing. https://www.sportsdietitians.com.au/factsheets/food-for-your-sport/boxing/..

[9] Thomas C, Langan-Evans C, Germaine M (2022). Case report: effect of low energy availability and training load on sleep in a male combat sport athlete. Front Sports Act Living.

[10] Blanton CA, Moshfegh AJ, Baer DJ, Kretsch MJ (2006). The USDA automated multiple-pass method accurately estimates group total energy and nutrient intake. J Nutr.

[11] Ainsworth BE, Haskell WL, Herrmann SD (2011). 2011 Compendium of Physical Activities: a second update of codes and MET values. Med Sci Sports Exerc.

[12] (2024). BIODYNAMICS: Basal metabolic rate. https://www.biodyncorp.com/product/310/bmr_310.html.

[13] Nelson KM, Weinsier RL, Long CL, Schutz Y (1992). Prediction of resting energy expenditure from fat-free mass and fat mass. Am J Clin Nutr.

[14] Nakamura K, Uchida M, Sato T (2021). Basic research on the primary prevention of boxing-related sports injuries with the development of a quantitative motion analysis software. J Phys Ther Sci.

[15] Januszko P, Lange E (2021). Nutrition, supplementation and weight reduction in combat sports: a review. AIMS Public Health.

[16] (2024). Analysis of body composition of Indian national level boxers and weightlifters: a comparative approach. https://www.eurchembull.com/issue-content/analysis-of-body-composition-of-indian-national-level-boxers-and-weightlifters-a-comparative-approach-10711..

[17] Tenforde AS, Barrack MT, Nattiv A, Fredericson M (2016). Parallels with the Female Athlete Triad in male athletes. Sports Med.

[18] Ghazzawi HA, Hussain MA, Raziq KM (2023). Exploring the relationship between micronutrients and athletic performance: a comprehensive scientific systematic review of the literature in sports medicine. Sports (Basel).

[19] Loucks AB, Verdun M, Heath EM (1998). Low energy availability, not stress of exercise, alters LH pulsatility in exercising women. J Appl Physiol (1985).

[20] Loucks AB, Thuma JR (2003). Luteinizing hormone pulsatility is disrupted at a threshold of energy availability in regularly menstruating women. J Clin Endocrinol Metab.

[21] Rauh MJ, Macera CA, Trone DW, Shaffer RA, Brodine SK (2006). Epidemiology of stress fracture and lower-extremity overuse injury in female recruits. Med Sci Sports Exerc.

[22] Wentz L, Liu PY, Ilich JZ, Haymes EM (2012). Dietary and training predictors of stress fractures in female runners. Int J Sport Nutr Exerc Metab.

[23] Ackerman KE, Holtzman B, Cooper KM (2019). Low energy availability surrogates correlate with health and performance consequences of relative energy deficiency in sport. Br J Sports Med.

[24] Malla H, Lal P (2022). Energy availability and macronutrient intake of elite Indian adolescent boxers. Int J Food Sci Nutr.

[25] Baranauskas M, Tubelis L, Stukas R (2018). Nutritional status and physical development of high-performance combat athletes in Lithuania. Balt J Sport Health Sci.

[26] Reale R, Burke LM, Cox GR, Slater G (2020). Body composition of elite Olympic combat sport athletes. Eur J Sport Sci.

[27] Durkalec-Michalski Durkalec-Michalski, Krzysztof Krzysztof, Sokołowski M (2016). Relationship between body composition indicators and physical capacity of the combat sports athletes. Archives of Budo.

[28] Tyagi P, Bhushanam GV (2021). A comparative study on the macronutrient intake of elite Indian female weightlifters and boxers. Int J Physiol Nutr Phys Educ.

[29] Vardardottir B, Gudmundsdottir SL, Tryggvadottir EA, Olafsdottir AS (2024). Patterns of energy availability and carbohydrate intake differentiate between adaptable and problematic low energy availability in female athletes. Front Sports Act Living.

